# Omarigliptin alleviates cognitive dysfunction in Streptozotocin-induced diabetic mouse

**DOI:** 10.1080/21655979.2022.2055699

**Published:** 2022-04-07

**Authors:** Xiaoyan Li, Ying Yin, Wenfeng Li, Shanshan Li, Dandan Zhang, Zehong Liu

**Affiliations:** Department of Endocrinology, Ganzhou People’s Hospital, Ganzhou, Jiangxi, China

**Keywords:** Omarigliptin, diabetes mellitus (DM), cognitive dysfunction, SIRT3, FOXO3a, mitochondrial function

## Abstract

Increasing epidemiological evidence supports the strong association between diabetes mellitus (DM) and cognitive dysfunction. Omarigliptin is a long-acting dipeptidyl peptidase 4 (DPP-4) inhibitor for the treatment of diabetes. However, the effect of Omarigliptin in diabetes-associated cognitive dysfunction has not been reported. In this study, we established an *in vivo* diabetic mice model through streptozotocin (STZ) treatment and investigated the therapeutic effect of Omarigliptin in diabetic mice. The results show that administration with Omarigliptin reduced the food and water intake of STZ-induced diabetic mice, accompanied by decreased blood glucose levels and increased serum insulin levels. The Y-Maze test demonstrated that Omarigliptin ameliorated cognitive dysfunction in STZ-induced diabetic mice. Omarigliptin presented a protective role in the brain, as shown by the decreased reactive oxygen species (ROS) level, increased NAD+/NADH ratio, adenosine triphosphate (ATP) level, and ATP synthase activity in the hippocampus. Omarigliptin induced the increased expression level of mitochondrial inner membrane protein sirtuin 3 (SIRT3) and regulated its substrates, including forkhead box O3a (FOXO3a) and superoxide dismutase 2 (SOD2). Furthermore, knockdown of SIRT3 abolished the protective effects of Omarigliptin on mitochondrial dysfunction and cognitive dysfunction in STZ-induced diabetic mice. Taken together, these findings suggest that Omarigliptin improved insulin sensitivity and cognitive function in STZ-induced diabetic mice. Mechanistically, SIRT3 expression is required for the effect of Omarigliptin. This study provided preclinical evidence that Omarigliptin has the neuroprotective effect to improve diabetes-associated cognitive dysfunction.

## Graphical abstract

**Figure:**
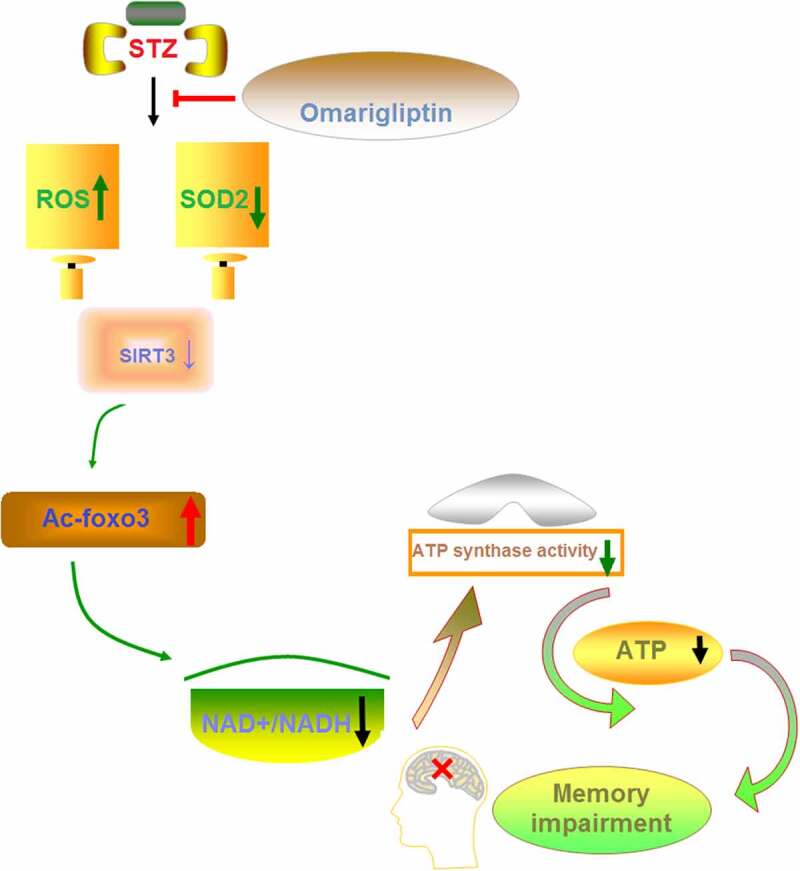

## 1. Introduction

Diabetes mellitus is a glucose metabolic disorder characterized by an elevated level of blood glucose resulting from progressive insulin deficiency [1]. Hyperglycemia results in macrovascular, microvascular, and neuropathic complications involving various organs, especially cardiovascular, renal, retinal, and peripheral nervous systems [2]. There are two types of diabetes mellitus, with type 2 being the most common diabetes and accounting for approximately 90% of diabetic patients, and type 1 affecting less than 10% of patients [3]. With the incidence rate increase, diabetes has become a major health burden in developing and developed countries [4].

People with diabetes often develop cognitive dysfunction including dementia and cognitive impairment, because high glucose levels cause brain impairment and induce sorbitol synthesis, leading to injury to blood vessel and nerves degeneration [5,6]. Hyperglycemia induces the production of reactive oxygen species (ROS), which are major contributors to neurodegeneration and neuronal oxidative damage [7,8]. Although accumulating evidence indicates that cognitive dysfunction is closely related to diabetes, there are no therapies that can manage the process of cognitive dysfunction at present.

Omarigliptin is an inhibitor of dipeptidyl peptidase-4 (DPP-4) developed for the treatment of diabetes [9]. It significantly improves glycemic levels due to its effective once-weekly pharmacokinetic profile [10]. Increasing evidence has shown that Omarigliptin possesses anti-inflammatory and anti-oxidative stress activities in high glucose conditions. It exerts anti-inflammatory and anti-insulin resistance properties in a pleiotropic manner in diabetic patients [10]. Omarigliptin ameliorates high glucose-induced inflammatory injury in renal glomerular endothelial cells through regulating the activation of the NOD-like receptor family pyrin domain containing 3 (NLRP3) inflammasome [11]. Omarigliptin shows its beneficial effects on diabetic patients through improving inflammation and insulin resistance [12]. It is efficacious for glucose variability and for controlling oxidative stress in diabetic patients [13]. Notably, Omarigliptin has the capacity to effectively pass through the BBB because of its lipophilic property and low molecular weight [14]. A recent study shows Omarigliptin attenuates lipopolysaccharide (LPS)-induced neuroinflammation and protects the integrity of the blood-brain barrier (BBB) against LPS via inhibiting the TLR4/Myd88/NF-κB signaling pathway [15]. These results suggest the potential repurposing of Omarigliptin for neurodegenerative diseases.

Sirtuins are a family of proteins involved in the regulation of metabolism and energy expenditure. Amongst the sirtuin family proteins, SIRT3 is localized primarily in the mitochondria [16]. The forkhead box class O (FOXO) family has been shown to regulate energy metabolism in many tissues. FOXO3A is a direct target of SIRT3 and functions as a transcriptional factor upon cellular stress. SIRT3 can directly deacetylate FOXO3A, which in turn increases the expression of downstream antioxidant proteins to alleviate oxidative stress [17]. The mitochondrial-dependent SIRT3-FOXO3A pathway has been shown to be involved in the pathogenesis of insulin resistance and diabetes mellitus [16].

We hypothesized that Omarigliptin could have a therapeutic effect on diabetes-associated neuro disorders. In the present work, we tested for the beneficial actions of Omarigliptin on cognitive dysfunction in streptozotocin (STZ)- treated mice and the roles of SIRT3 protein on the protective effects of Omarigliptin in the brain.

## 2. Materials and methods

### 2.1 Animals

**Figure 1.**
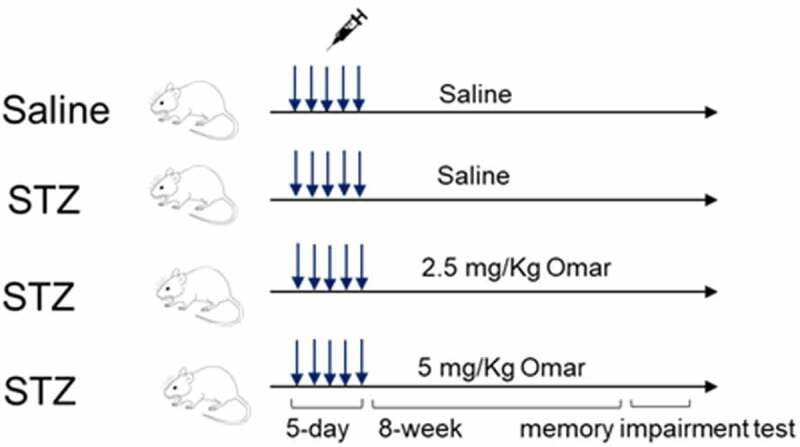
Representative diagram of experimental protocols.

The animal studies were approved by the Ethical Committee of Ganzhou People’s Hospital (No. GPHE-2019-0322) and conducted according to the Guidelines for the Care and Use of Experimental Animals. Forty C57BL/6 male mice (12-week old) were housed in a standard laboratory condition as described in another study [18]. Animals were randomly divided into four groups (n = 10): Sham group, 0.5 ml saline intraperitoneal injection (I.P.) daily for five days; diabetic model group, 50 mg/kg STZ in 0.5 ml saline I.P. daily for five days [19]; Omarigliptin low dosage (2.5 mg/kg) treatment group, 50 mg/kg STZ in 0.5 ml saline I.P. daily for five days and orally administered with 2.5 mg/kg/week Omarigliptin for 8 weeks; Omarigliptin high dosage (5 mg/kg) treatment group, 50 mg/kg STZ in 0.5 ml saline I.P. daily for five days and orally administered with 5 mg/kg/week Omarigliptin for 8 weeks. The food and water intake were recorded. After experiments, all mice were anesthetized by inhalation with 2–4% isoflurane, and then exsanguination was performed by cardiac puncture. The blood samples were collected for blood glucose detection and for the preparation of plasma. A representative diagram of experimental protocols is shown in Figure 1. Hippocampus was rapidly collected and stored at −80°C. Biochemical indexes including blood glucose and serum insulin levels were measured using commercial kits (Nanjing Jian Cheng Bioengineering Institute, Nanjing, China).

### 2.2 SIRT3 knockdown

Lentiviral vector encoding SIRT3 targeted shRNA (LV-shSIRT3) and a lentiviral vector encoding negative control shRNA (LV-shNC) were synthesized and packaged by GenePhama company (Shanghai, China). Fifty C57BL/6 male mice (12-week old) were divided into five groups: Sham group, 0.5 ml saline I.P. daily for five days; negative control group, intracerebroventricularly injected with LV-shNC; diabetic model group, 50 mg/kg STZ in 0.5 ml saline I.P. daily for five days; Omarigliptin treatment group, 50 mg/kg STZ in 0.5 ml saline I.P. daily for five days and orally administered with 5 mg/kg/week Omarigliptin for 8 weeks; SIRT3 knockdown group, except for 50 mg/kg STZ + 5 mg/kg/week Omarigliptin treatment, the mice were intracerebroventricularly injected with LV-shSIRT3. The animal samples were collected and prepared as described above.

### 2.3 Y-maze test

Y-maze test was performed as previously described [20] to assess spatial working memory. The Y-maze apparatus was comprised of three black plastic arms (10 × 10 × 10 cm^3^) connected with passages (4 × 5 cm). The test was continued for two days and each mouse was examined 10 times per day. The first day was used for the learning trial. On the second day (formal testing trial), the number of correct choices was recorded manually 10 times.

### 2.4 Measurement of ROS level and SOD activity

Brain hippocampus tissues were cut into 4.0-μm sections, followed by placed on chilled microscope slides for the determination of ROS production as previously described [21]. The images of the tissue were recorded with an automatic fluorescence microscope (BX63, Olympus Optical Ltd., Tokyo, Japan). The ROS levels were quantified with Image J software (NIH, USA). The brain tissue was dissected on ice and then used for the preparation of tissue homogenate. Afterward, the brain SOD activity was measured using a SOD assay kit (Jian Cheng Bioengineering Institute), following the manufacturer’s directions.

### 2.5. Reduced Glutathione (GSH) Concentrations

50 μg supernatants of hippocampal tissue from each experimental group were deproteinized by adding 2% metaphosphoric acid. After centrifugation at 7000 × g for 10 min, the supernatant was collected and added to o-phthaldialdehyde (1 mg/mL in methanol) for 15 min in a dark room at room temperature. Fluorescent intensity was recorded using excitation and emission wavelengths of 350 and 420 nm, respectively.

### 2.6 Measurement of ATP and ATP synthase (ATPase) activity

The ATP content and ATPase activity in brain hippocampus tissue homogenate were measured by colorimetric method using ATP assay kit and ATPase assay kit, respectively (Nanjing Jian Cheng Bioengineering Institute).

### 2.7 NAD^+^/NADH ratio assay

NAD^+^/NADH ratio in brain hippocampus tissue homogenate was measured using the NAD^+^/NADH Quantification Colorimetric Kit (Cell Biolabs, Inc., San Diego, CA) according to the manufacturer’s instructions.

### 2.8 Western blot analysis

Total protein was extracted from the brain hippocampus tissues of mice in different groups for the determination of SOD2, SIRT3, and Ac-FOXO3a/FOXO3a. After separation by 10% SDS-polyacrylamide gels, proteins were transferred onto PVDF membranes, followed by incubation with primary antibodies and secondary antibody. The primary antibodies against SOD2, SIRT3, Ac-FOXO3a, FOXO3a, and β-actin (Abcam, Cambridge, MA, USA) were diluted in 1: 500 and incubated for 8 h at 4°C, while secondary antibody (Abcam) was diluted in 1: 3000 and incubated for 1 h at 37°C.

### 2.9 RNA isolation and qRT-PCR

Total RNA in brain hippocampus tissues was purified using a total RNA kit (TaKaRa Bio, Shiga, Japan), and cDNA was produced using a First Strand cDNA Synthesis Kit (TaKaRa). The resulting cDNA was amplified by PCR using SYBR Green qPCR Master Mix (Bio-Rad Laboratories, Hercules, CA, USA). The mRNA level of SIRT3 was calculated by the ΔΔC_T_-method relative to GAPDH [22]. PCR primers used in this study were purchased from a commercial source (Origene, USA). The sequences of primers include: SIRT-3 Upstream 5’-GCTACATGCACGGTCTGTCGAA-3’, SIRT-3 Downstream: 5’-CAATGTCGGGTTTCACAACGCC-3’; GAPDH Upstream 5’-CATCACTGCCACCCAGAAGACTG-3’, GAPDH Downstream, 5’-ATGCCAGTGAGCTTCCCGTTCAG-3’.

### 2.10 Statistical Analysis

Data are presented as the mean ± standard deviation (S.D.) from three independent experiments. Differences were considered as significant at *p* < 0.05. We used one-way analysis of variance (ANOVA) with Tukey’s post hoc test to analyze differences with GraphPad Prism 5.0 (GraphPad Software for Science, San Diego, CA).

## 3. Results

In this study, we established a diabetic mouse model through Streptozotocin (STZ) treatment and displayed that administration of Omarigliptin had a protective effect in diabetic mice. We showed that Omarigliptin therapy reduced the food and water intake, and increased insulin sensitivity in diabetic mice. Behavior tests showed that Omarigliptin improved insulin sensitivity and cognitive function in diabetic mice. We showed that Omarigliptin treatment ameliorated diabetes-induced mitochondrial dysfunction in the brain, and the expression of SIRT3 was required for the effect of Omarigliptin.

### 3.1 Effect of Omarigliptin on glucose and insulin levels in STZ-induced diabetic mice

**Figure 2.**
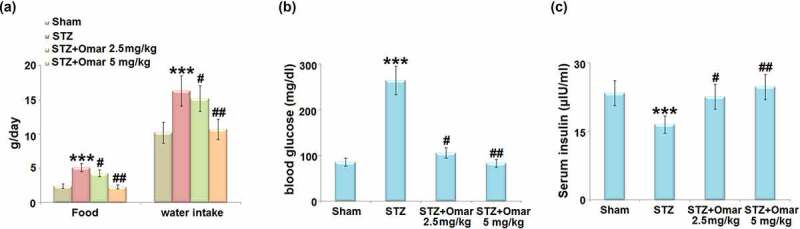
The effect of Omarigliptin on STZ-challenged diabetic mice. (a) Food and water intake; (b) blood glucose; (c) Serum insulin (***, P < 0.001 vs. vehicle group; #, ##, P < 0.05, 0.01 vs. STZ group).

The food and water intake in the diabetic model group were markedly increased relative to the sham group. Administration of Omarigliptin (2.5 or 5 mg/kg) reduced the food and water intake (Figure 2(a)). The blood glucose was significantly elevated, while the serum insulin level was significantly decreased in the diabetic model group. Whereas, Omarigliptin administration (2.5 or 5 mg/kg) caused a significant decrease in blood glucose, accompanied by increased serum insulin levels (Figure 2(b,c)).

### 3.2. Effect of Omarigliptin on cognitive dysfunction in STZ-induced diabetic mice

**Figure 3.**
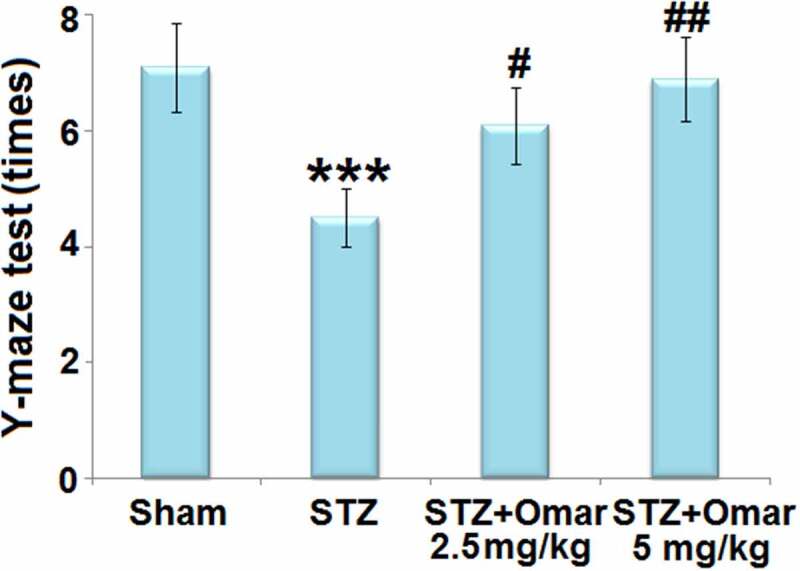
Omarigliptin treatment improves Y-maze test of STZ-challenged diabetic mice. Y-maze test was shown (***, P < 0.001 vs. vehicle group; #, ##, P < 0.05, 0.01 vs. STZ group).

We first assessed the cognition of mice in multiple groups using a Y-Maze test. As shown in Figure 3, the mice in the diabetic model group showed a significant decrease in the number of correct times as compared to the sham group. Mice in the Omarigliptin administration (2.5 or 5 mg/kg) groups showed a significant increase in the number of correct times compared to the diabetic model group, indicating that Omarigliptin (2.5 or 5 mg/kg) improved cognitive dysfunction in STZ-induced diabetic mice.

### 3.3 Effect of Omarigliptin on oxidative stress in the STZ-induced diabetic mice

**Figure 4.**
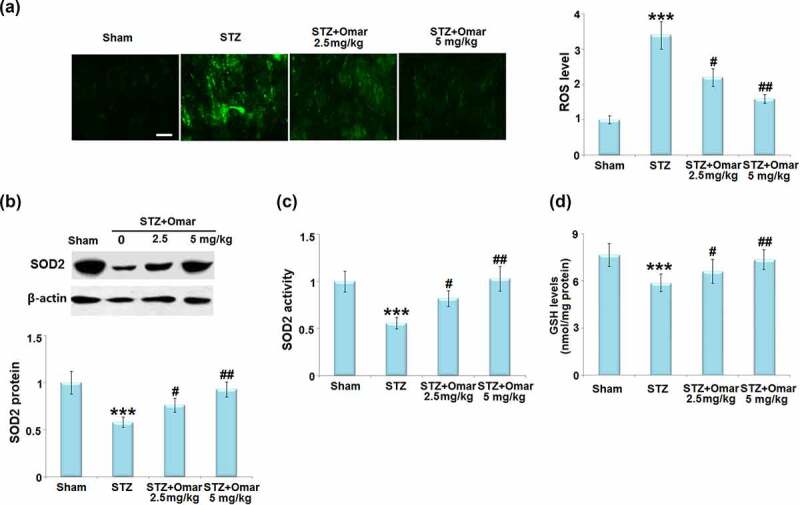
Effect of Omarigliptin on oxidative stress in the brains of STZ-challenged diabetic mice. (a). ROS level; Scale bar, 100 μm; (b). SOD2 protein expressions; (c). SOD2 activity; (d). The levels of reduced GSH (***, P < 0.001 vs. vehicle group; #, ##, P < 0.05, 0.01 vs. STZ group).

To evaluate the effect of Omarigliptin on STZ-induced oxidative stress in brain hippocampus tissues, the ROS level, SOD2 protein expressions, and SOD2 activity were measured. ROS level in the brain was significantly higher in the diabetic model group than in the sham group. After treatment with Omarigliptin (2.5 or 5 mg/kg), brain ROS levels were markedly decreased by 35.3% and 52.9%, respectively (Figure 4(a)). Western blot analysis showed that the SOD2 expression level was reduced by 42% in the diabetic model group when compared with the sham group. After treatment with 2.5 or 5 mg/kg Omarigliptin, the expression level of SOD2 was upregulated by 24.0% and 60.3%, respectively (Figure 4(b)). Likewise, the decreased SOD2 activity in the diabetic model group was also respectively increased by 46.2% and 83.9% after 2.5 or 5 mg/kg Omarigliptin treatment (Figure 4(c)). Importantly, the STZ challenge significantly decreased the levels of GSH in the hippocampus, which was rescued by Omarigliptin treatment in a dose-dependent manner (Figure 4(d)).

### 3.4 Effect of Omarigliptin on SIRT3 and FOXO3a expression in the STZ-induced diabetic mice

**Figure 5.**
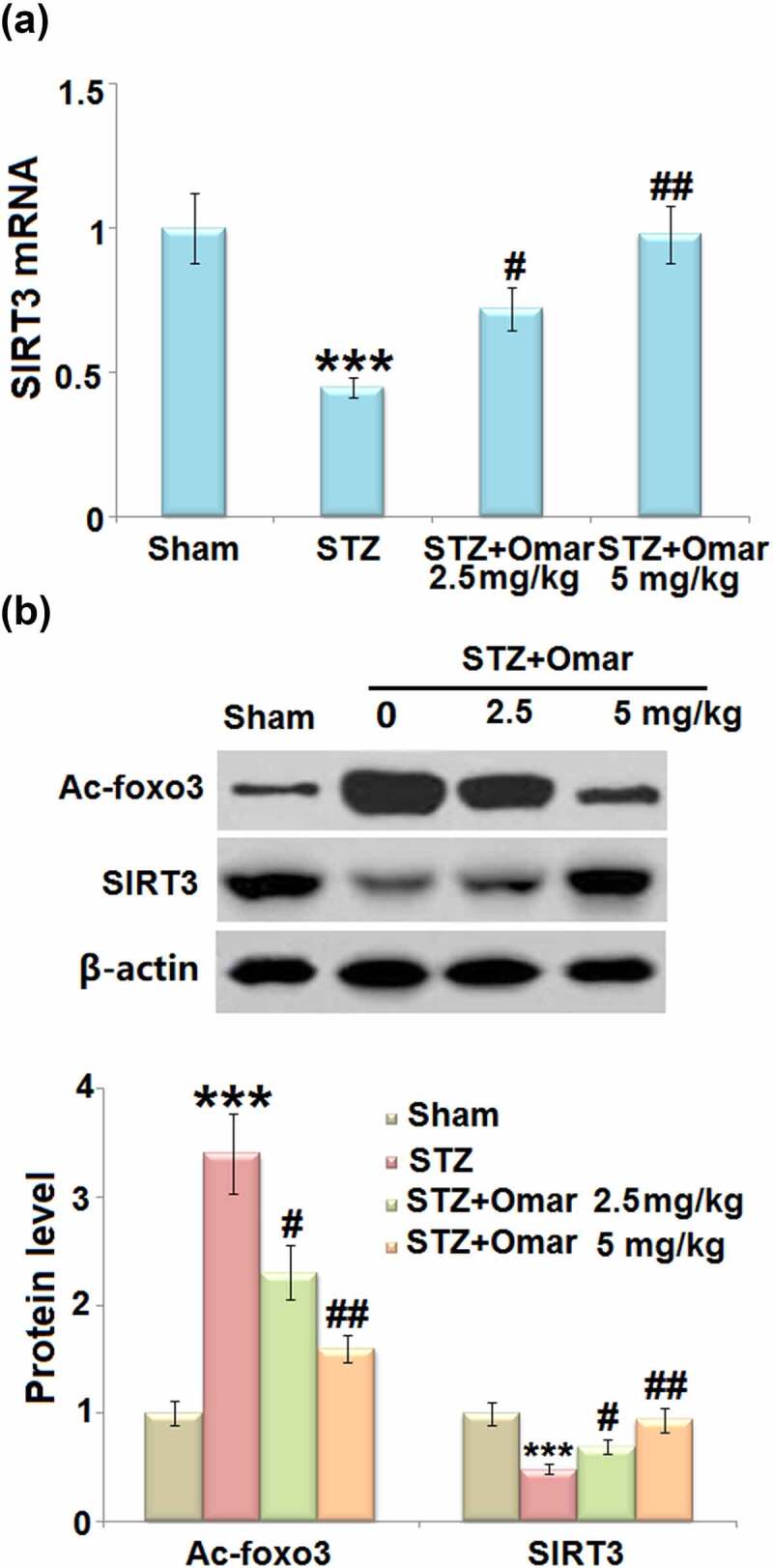
The effect of Omarigliptin on the expression of Ac-foxo3a and SIRT3 in the brain of STZ-challenged diabetic mice. (a) mRNA level of SIRT3; (b) Protein level of Ac-foxo3 and SIRT3 (***, P < 0.001 vs. vehicle group; #, ##, P < 0.05, 0.01 vs. STZ group).

Given that FOXO3a and SIRT3 are involved in the development of diabetes, we investigated how Omarigliptin affected their gene expressions in brain hippocampus tissues. The mRNA level of SIRT3 was decreased by 55% in STZ-induced diabetic mice compared to mice in the sham group. Omarigliptin administration (2.5 or 5 mg/kg) upregulated the mRNA level of SIRT3 by 60.0% and 117.8% relative to the diabetic model group (Figure 5(a)). Western blot showed that STZ-induced diabetic mice exhibited an increased Ac-FOXO3a level and decreased SIRT3 expression level. However, Ac-FOXO3a was significantly decreased, while the SIRT3 expression level was increased in the Omarigliptin- administrated groups (Figure 5(b)). The results indicate that Omarigliptin induced increased SIRT3 expression and prevented the acetylation of FOXO3a.

### 3.5 Effect of Omarigliptin on NAD^+^/NADH in STZ-induced diabetic mice

**Figure 6.**
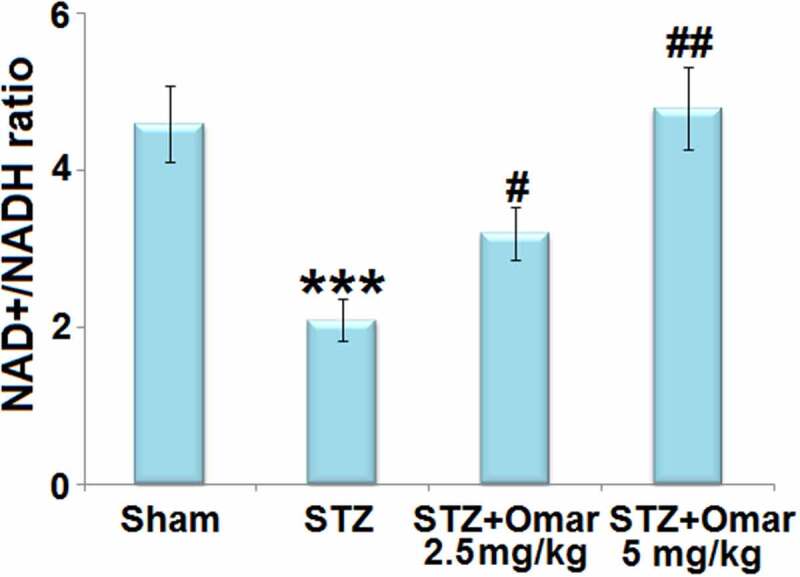
The effect of Omarigliptin on the expression of NAD+, NADH, and NAD+/NADH in the brain of STZ-challenged diabetic mice. NAD+/NADH ratio in hippocampus (***, P < 0.001 vs. vehicle group; #, ##, P < 0.05, 0.01 vs. STZ group).

Noteworthy, we found that STZ treatment brought about a significant decrease (0.46-fold) of the NAD^+^/NADH ratio in mice hippocampus tissues. Whereas, Omarigliptin administration (2.5 or 5 mg/kg) respectively led to a 1.52- or 2.29-fold increase in the NAD^+^/NADH ratio compared to the STZ-induced diabetic mice (Figure 6).

### 3.6 Effect of Omarigliptin on the ATP level and ATP synthase activity in STZ-induced diabetic mice

**Figure 7.**
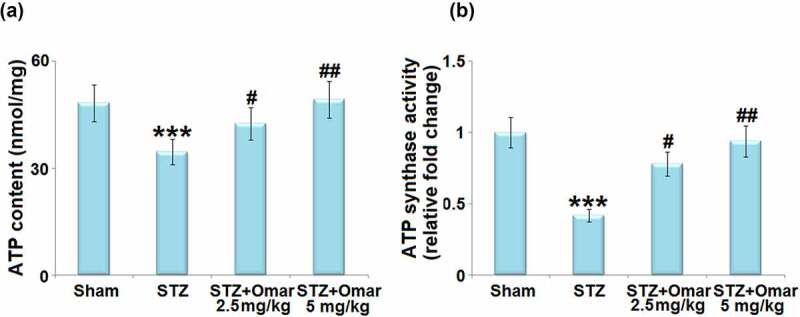
The effect of Omarigliptin on the levels of ATP and ATP synthase activity in the brain of STZ-challenged diabetic mice. (a) ATP content; (b) ATP synthase activity (***, P < 0.001 vs. vehicle group; #, ##, P < 0.05, 0.01 vs. STZ group).

As shown in Figure 7(a), ATP content was reduced in STZ-induced diabetic mice relative to control mice. Mice administrated with Omarigliptin (2.5 or 5 mg/kg) were found to present higher ATP content than that in the hippocampi of the diabetic model group. Meanwhile, STZ induction produced a decrease in ATP synthase activity, which could be attenuated by Omarigliptin (2.5 or 5 mg/kg) treatment (Figure 7(b)).

### 3.7 Involvement of SIRT3 in Omarigliptin-induced neuroprotection

**Figure 8.**
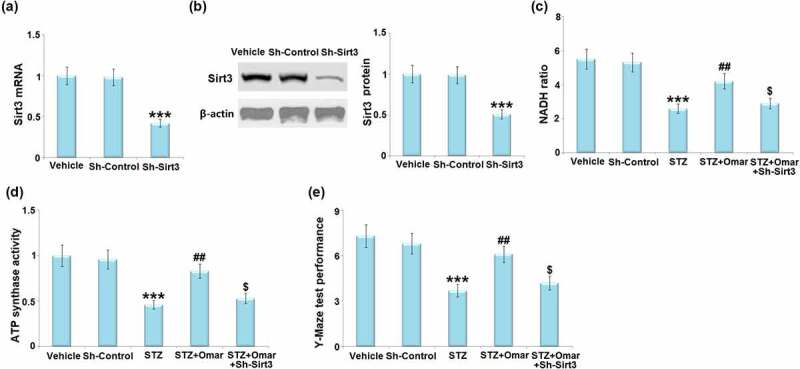
Involvement of Sirt3 in Omarigliptin-induced neuroprotection against memory damage in STZ-challenged diabetic mice. Mice were intracerebroventricularly injected of Sirt3-targeted shRNA (Sh-Sirt3) or control shRNA (Sh-Control), (a) the expression of Sirt3 mRNA; (b) and protein of Sirt3; (c)NAD+/NADH ratio; (d) ATP synthase activity; (e). Y-maze test performance (***, P < 0.001 vs. vehicle group; ##, P < 0.01 vs. STZ group; $, P < 0.01 vs. STZ+ Omarigliptin group).

To confirm the involvement of SIRT3 in the neuroprotective effect of Omarigliptin on STZ-induced diabetic mice, LV-shSIRT3 was intracerebroventricularly injected into the mice to knock down SIRT3. Figure 8(a,b) show that the mRNA and protein levels of SIRT3 were markedly downregulated by 58% and 49% after injection with LV-shSIRT3, as compared to the mice injected with LV-shNC. The Omarigliptin-caused increase in the NAD^+^/NADH ratio and ATP synthase activity in STZ-induced diabetic mice were prevented by knockdown of SIRT3 (Figure 8(c,d)). In addition, the Omarigliptin-caused improved performance in the Y-Maze test was abolished in the SIRT3- knockdown group (Figure 8(e)).

## 4. Discussion

There is increasing epidemiological evidence for a strong association between diabetes and cognitive dysfunction in animal models and humans [22]. Specifically, the data show that diabetes is associated with a 50% increase in the risk for cognitive dysfunction [23]. In the present study, we found that cognitive dysfunction was observed in STZ-induced diabetic mice, which could be improved by Omarigliptin treatment, accompanied by decreased blood glucose levels and increased serum insulin levels. Previous studies have shown that hyperglycemia-induced mitochondrial ROS formation may lead to mitochondrial dysfunction and mitochondrial oxidative stress [24]. Besides, hyperglycemia induces the calpain-1 expression in the mitochondria and cleaves the ATP synthase α subunit, resulting in reduced ATP synthase activity [25]. The impairment in the activity of ATP synthase also affects mitochondrial function, which may result in energy failure. Since the brain tissue is a dynamic organ that requires a great deal of energy for maintaining its normal function, and an essential role of mitochondria is to produce ATP, the consequence of severe mitochondrial dysfunction is a crucial factor for cognitive function [26]. Our results prove that brain ROS levels were increased, whereas the NAD^+^/NADH ratio, ATP level, and ATP synthase activity were decreased in STZ-induced diabetic mice, implying that STZ caused mitochondrial dysfunction and oxidative damage. However, Omarigliptin presented a protective role in mitochondrial dysfunction and oxidative damage.

Sirtuins (SIRTs) are a family of NAD^+^-dependent protein deacetylases, which can be activated in response to low cellular energy stores [27]. SIRTs have been implicated in regulating a series of physiological intracellular processes [28]. SIRT3 is a mitochondrial inner membrane protein and regulates mitochondrial function and metabolism in response to multiple types of cellular damage, including oxidative damage [29]. In this study, we found that Omarigliptin administration upregulated the expression of SIRT3 at both mRNA and protein levels in STZ-induced diabetic mice. The results indicate that Omarigliptin might exert its protective role via regulating SIRT3.

FOXO3a, a forkhead transcription factor, is a mitochondrial protein that mediates the expression of multiple genes governing cellular development, survival, differentiation, autophagy, apoptosis, and stress resistance [30,31]. FOXO3a is a SIRT3 substrate that interacts with SIRT3 in mitochondrial extracts. SIRT3 has been shown to mediate the deacetylation of FOXO3a, thereby reducing intracellular ROS production by upregulating the antioxidant enzymes [32]. In addition, SOD2, an enzyme responsible for converting superoxide to molecular oxygen or hydrogen peroxide, is a substrate of SIRT3 [33]. The binding of SIRT3 with SOD2 causes the deacetylation and activation of SOD2, thereby reducing the ROS level [34]. Based on these findings, we can see that the SIRT3 signaling cascade is crucial for maintaining mitochondrial homeostasis and cellular function through regulating intracellular ROS levels. We demonstrated that Omarigliptin inhibited the STZ-induced acetylation of FOXO3a and induced the activation of SOD2, which might be attributed to the induction of SIRT3. Furthermore, knockdown of SIRT3 abolished the protective effects of Omarigliptin on mitochondrial and cognitive dysfunction in STZ-induced diabetic mice. These findings suggest that Omarigliptin exerted its positive effects via regulating SIRT3.

## 5. Conclusion

In conclusion, this study shows that the DPP-4 inhibitor Omarigliptin improved insulin sensitivity and cognitive function in STZ-induced diabetic mice. Omarigliptin treatment ameliorated diabetes-induced mitochondrial dysfunction and oxidative stress, and SIRT3 was required for the effects of Omarigliptin. This study provides preclinical evidence that Omarigliptin has a neuroprotective effect in improving diabetes-associated cognitive dysfunction.

## Availability of data materials

Requests for data and materials should be addressed to the corresponding author.
